# Analysis of genetic characteristics of 436 children with dysplasia and detailed analysis of rare karyotype

**DOI:** 10.1515/biol-2022-0046

**Published:** 2022-04-26

**Authors:** Zong-Yu Miao, Shi-Feng Chen, Hong Wu, Xiao-Yan Liu, Hui-Yuan Shao

**Affiliations:** Medical Laboratory, Yantai Yu Huang Ding Hospital, 20#, The East Road of Yu Huang Ding, Zhifu District, Yantai, 264000, Shandong, China

**Keywords:** dysplasia, trilogy 21, 4q-syndrome, monomer 9p, 18q-syndrome

## Abstract

Chromosomal abnormality is one of the important causes of dysplasia in children. However, due to regional and ethnic differences, the reported rates of chromosomal abnormalities in patients with dysplasia vary greatly. Moreover, the clinical manifestations in children with rare chromosomal diseases were heterogeneous. So, we retrospectively analyzed the karyotype results of 436 children with dysplasia and conducted a detailed analysis of rare chromosomal diseases. The results showed that chromosomal abnormalities were present in 181 of 436 cases. Intellectual disability, dysmorphology, congenital malformations, the disorder of sexual development, and short stature were the main five clinical symptoms in children with chromosomal abnormalities. Moreover, 136 cases of Trisomy 21 (Tri21) were detected, of which 130 were standard Tri21, 5 were robertsonian Tri21, and 1 was chimera type. In addition, 16 cases of rare abnormal karyotype, including complex Tri21, complex Turner syndrome, 4p-syndrome, 18q-syndrome, and 5p-syndrome, were also detected. In summary, chromosome abnormality is one of the important causes of dysplasia in children. Furthermore, prenatal screening and diagnosis could play a great significance in preventing dysplasia in children. In addition, the retrospective analysis of rare cases is valuable for clinical diagnosis and risk assessment of recurrence.

## 1 Introduction

Dysplasia in children is increasingly becoming a prominent public health and social problem. Dysplasia in children affects children’s health and quality of life and the healthy and sustainable development of society. So, reducing congenital disabilities has great social significance. Therefore, it is necessary to delve into the causes of dysplasia in children.

Congenital heart disease (CHD) is one of the most common dysplasias in children. The incidence of CHD in neonates is 8–9/1,000, and nearly 1.35 million CHD neonates are born every year in the world [1]. Chromosomal causes of CHD include chromosome aneuploidies, such as Trisomy 21 (Tri21). Chromosomal aneuploidies represent 12.5% of CHD causes [2]. In addition, Tri21 syndrome, a common chromosomal disorder, is the most common cause of severe mental retardation, special facial features, and abnormal bone development in children.

Disorder of sexual development (DSD) and short stature are also the most common dysplasia symptoms in children. Sex chromosome abnormality is one of the most common causes of DSD and short stature. Common sex chromosomal disorders include Klinefelter syndrome (1/1,000–1/2,000 in male newborns), Turner syndrome (TS, approximately 1/5,000 in newborn girls), XYY syndrome (1/900 in males), and XXX syndrome (1/1,000 in newborn girls). Patients with sex chromosome diseases are often accompanied by gonadal dysplasia, secondary sexual signs dysplasia, fertility disorders, mild mental abnormalities, mental deficiencies, and other symptoms. In addition, children with TS often have short stature. Children with severe short stature are vulnerable to diverse developmental, social, and educational problems [3]. Therefore, the abnormality of the sex chromosome is one of the important causes of dysplasia in children.

There is a significant evidence in the literature that the chromosome partial monomer or trisomy can lead to severe dysplasia in children. The clinical examination of the patients with structurally abnormal chromosome 15 revealed a series of clinical symptoms, such as intellectual disability, CHD, severe growth retardation, hypertelorism, microcephalus, dysmorphology, and multiple sclerosis hyperpigmented or café-au-lait spots, short stature, and clinodactyly [4,5,6,7]. The common clinical features of the patients with partial trisomy of chromosome 8 included developmental delay, intellectual disability, mental retardation, severe hypotonia, hypospadias, skeletal anomalies, renal dysplasia, attention-deficit hyperactivity disorder, atypical facial appearance, and congenital hypoplasia of the tongue [8,9,10,11,12,13]. The short arm monosomy of chromosome 9 may present developmental delay, hypotonia, trigonocephaly, psychomotor developmental delay, learning difficulties, trigonocephaly, facial dysmorphia, and genital abnormalities [14,15].

Thus, the chromosomal abnormality is one of the important causes of dysplasia in children, which seriously endangers children’s physical and mental health and brings heavy spiritual and economic burdens to families and society. However, due to regional and ethnic differences, the reported rates of chromosomal abnormalities in patients with dysplasia vary greatly. So, we retrospectively analyzed the karyotype results of 436 children with dysplasia and conducted a detailed analysis of rare cases to clarify the chromosomal abnormality rate and distribution of abnormal chromosomes in local children with dysplasia and provide a theoretical basis for clinical diagnosis and prenatal diagnosis, and assess the risk of recurrence.

## 2 Patients and methods

### 2.1 Patients

Four hundred and thirty-six cases (359 male and 77 female) in children with clinically diagnosed dysplasia in the affiliated Yantai Yu Huang Ding Hospital of Qingdao University Medical College from January 1, 2012 to October 31, 2019, were recruited. The age group of the participants ranged from 1 day to 16 years. Patients with dysplasia caused by autoimmune, chemo- or radio-therapy, infectious, and iatrogenic injury were excluded. The clinical data of confirmed cases were retrospectively collected and analyzed.


**Informed consent:** Informed consent has been obtained from all individuals included in this study.
**Ethical approval:** The research related to human use has been complied with all the relevant national regulations, institutional policies, and in accordance with the tenets of the Helsinki Declaration and has been approved by the Ethical Committee of Yantai Yu Huang Ding Hospital.

### 2.2 G banding

Standard chromosomal analyses with G banding were performed on routinely cultured peripheral blood lymphocytes [16]. Thirty metaphases per patient were counted, and a minimum of five metaphases were analyzed. For the chimeric case, at least 100 metaphases were counted. Chromosome polymorphisms, such as pericentric inversion of chromosome 9, centromeric heterochromatin variants, and satellite variants, were classified as normal. The karyotypic reports were based on the International System for Human Cytogenetic Nomenclature.

## 3 Results

Chromosomal abnormalities were detected in 181 of 436 cases. The abnormal rate was 41.51%. Among them were 153 cases of autosomal abnormality (84.53%, 153/181, Table 1) and 28 cases of a sex chromosome abnormality (15.47%, 28/181, Table 2). The main clinical symptoms of 181 children with chromosomal abnormalities were intellectual disability (80.66%, 146/181), dysmorphology (71.82%, 130/181), congenital malformations (22.10%, 40/181), DSD (12.15%, 22/181), and short stature (8.29%, 15/181).

**Table 1:** Distribution of autosomal abnormalities in children with dysplasia

Classification	Chromosome karyotypes	Number of cases	Constituent ratio (%)	Abnormality rate (%)
**Numerical abnormality**		**132**	**86.27**	**30.27**
	47,XX,+21	40	26.14	9.17
	47,XY,+21	86	56.21	19.72
	47,XX,+21[12]/46,XX[177]	1	0.65	0.23
	47,XY,+21,inv(9)(p11q12)	1	0.65	0.23
	47,XY,+21,13pstkstk	1	0.65	0.23
	47,XX,+21,inv(9)(p11q13)	1	0.65	0.23
	47,X,Yqs,+21	1	0.65	0.23
	47,XX,+mar[74]/46,XX[26]	1	0.65	0.23
			
**Structural abnormality**		**21**	**13.73**	**4.82**
	46,XX,der(13;14)(q10;q10),+21	1	0.65	0.23
	46,XY,der(14;21)(q10;q10),+21	2	1.31	0.46
	46,XX,der(21;21)(q10;q10),	1	0.65	0.23
	t(1;12)(q43;p12.1),inv(15)(q13q24)			
	46,XX,der(21;21)(q10;q10)	1	0.65	0.23
	46,XY,del(4)(q33)	1	0.65	0.23
	46,XY,del(5)(p14)	1	0.65	0.23
	46,XY,del(5)(p14.3)	1	0.65	0.23
	46,XX,del(13)(q31)	1	0.65	0.23
	46,XX,del(8)(p23.1)	1	0.65	0.23
	46,XX,add(16)(q24)	1	0.65	0.23
	46,XY,r(9)(p24q34)	1	0.65	0.23
	46,XY,der(9)t(2;9)(p25;p22)mat	1	0.65	0.23
	46,XY,der(9)t(7;9)(p15;p22)pat	1	0.65	0.23
	45,XX,der(15;21)(q10;q10)mat,del(18)(q21)	1	0.65	0.23
	46,XX,der(3)del(3)(p21.3p23)t(2;3)			
	(q11.2;p23)	1	0.65	0.23
	46,XX,t(9;15)(p24;q13)	1	0.65	0.23
	46,XX,t(9;16)(q13;q22)	1	0.65	0.23
	46,XX,t(2;8)(q13;q23)	1	0.65	0.23
	46,XX,t(1;3)(p22.1;q27)	1	0.65	0.23
	46,XX,inv(12)(p12.2q15)	1	0.65	0.23
**Combination**		**153**	**100**	**35.09**

**Table 2:** Distribution of sexual chromosomal abnormalities in children with dysplasia

Classification	Chromosome karyotypes	Number of cases	Constituent ratio (%)	Abnormality rate (%)
**Numerical abnormality**		**10**	**35.71**	**2.29**
	45,X	7	25.00	1.61
	45,X[73]/47,XXX[27]	1	3.57	0.23
	45,X[78]/47,XXX[22]	1	3.57	0.23
	45,X[90]/46,XX[6]	1	3.57	0.23
**Structural abnormality**		**16**	**57.15**	**3.67**
	46,X,i(X)(q10)	5	17.87	1.15
	45,X[91]/46,X,i(X)(q10)[9]	1	3.57	0.23
	46,X,i(X)(q10)[89]/45,X[11]	1	3.57	0.23
	45,X[61]/46,X,i(X)(p11.3)[39]	1	3.57	0.23
	46,X,idic(Y)(q11.22)[94]/45,X[6]	1	3.57	0.23
	45,X,inv(9)(p11q13)[72]/46,X,dic(Y)(q11.23),inv(9)(p11q13)[10]/46,XY, inv(9)(p11q13)[4]	1	3.57	0.23
	46,X, idic(Y)(q11.23)[44]/45,X[56]	1	3.57	0.23
	45,X[120]/46,X,r(X)(p22.2q22.2)[12]/	1	3.57	0.23
	46,X,rdup(X)(p22.2q22.2)[4]			
	45,X[80]/46,X,idic(X)(p11.3)[20]	1	3.57	0.23
	46,X,idic(X)(p11.2)[92]/45,X[9]/	1	3.57	0.23
	47,X,idic(X)(p11.2),idic(X)(p11.2)[5]			
	46,X,idic(X)(p11.2)[90]/45,X[10]	1	3.57	0.23
	46,X,idic(X;X)(q21.3;q11.1)	1	3.57	0.23
	46,XX (male)	**2**	**7.14**	**0.46**
**Sex reversal combination**		**28**	**100**	**6.42**

One hundred and thirty-six cases of Tri21 were detected (31.19%, 136/436), including 89 male and 47 female, with a ratio of 1.89:1. Among them, 130 cases were standard Tri21 (95.59%, 130/136), 5 cases were Robertsonian Tri21 (3.79%, 5/132) and 1 case was a chimeric type (0.74%, 1/136).

Twenty-eight cases of sex chromosome abnormalities were detected, among which 20 cases were TS (7 cases were 45,X, 13 cases were 45,X mosaic), 5 cases were 46,X,i(X)(q10), 2 cases were sexual reversal, and 1 case was 46,X,idic(X;X)(q21.3;q11.1).

**Figure 1:**
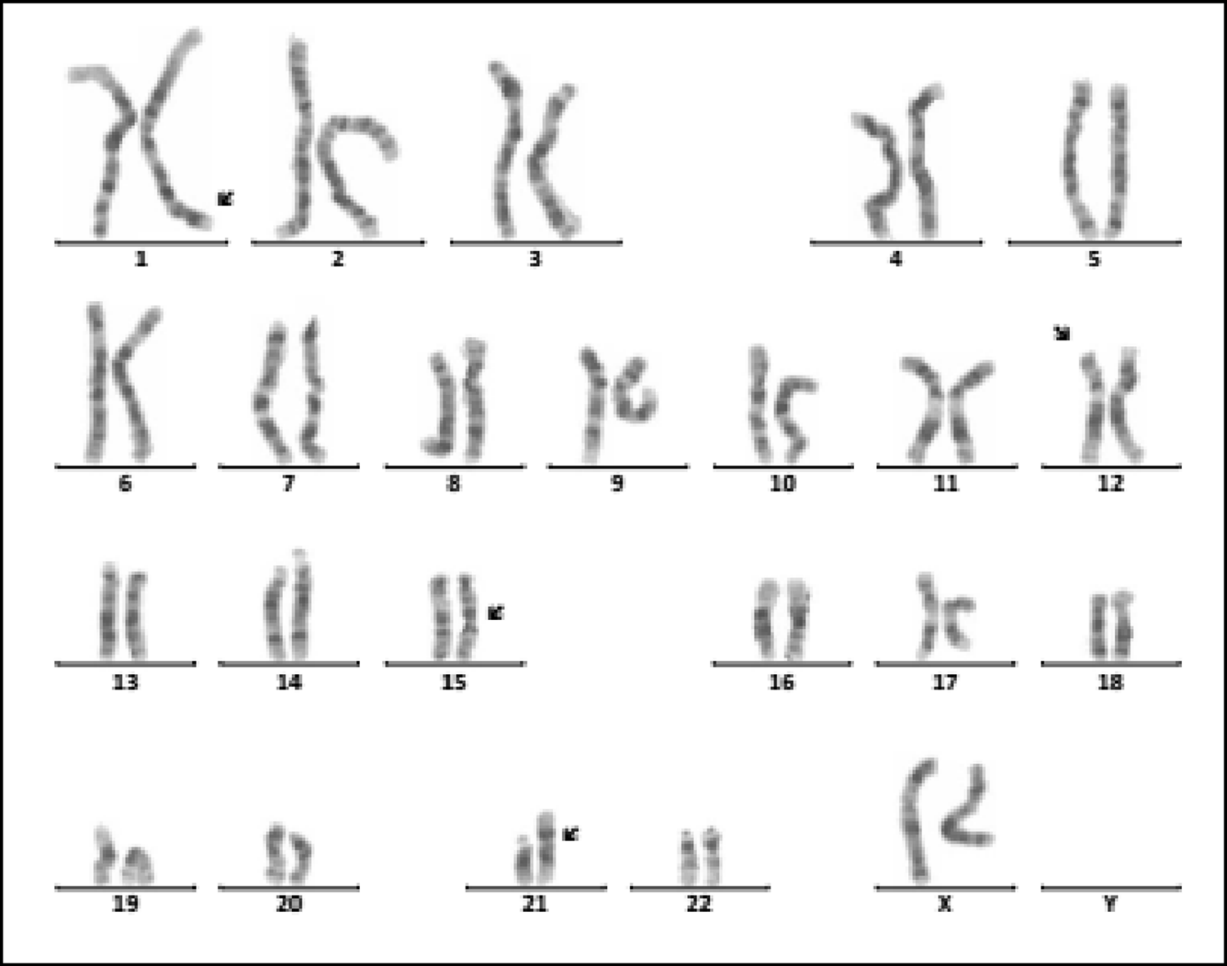
Karyotype of 46,XX, der(21;21)(q10;q10),t(1;12)(q43;p12.1),inv(15)(q13q24). The arrows show abnormal chromosomes.

**Figure 2:**
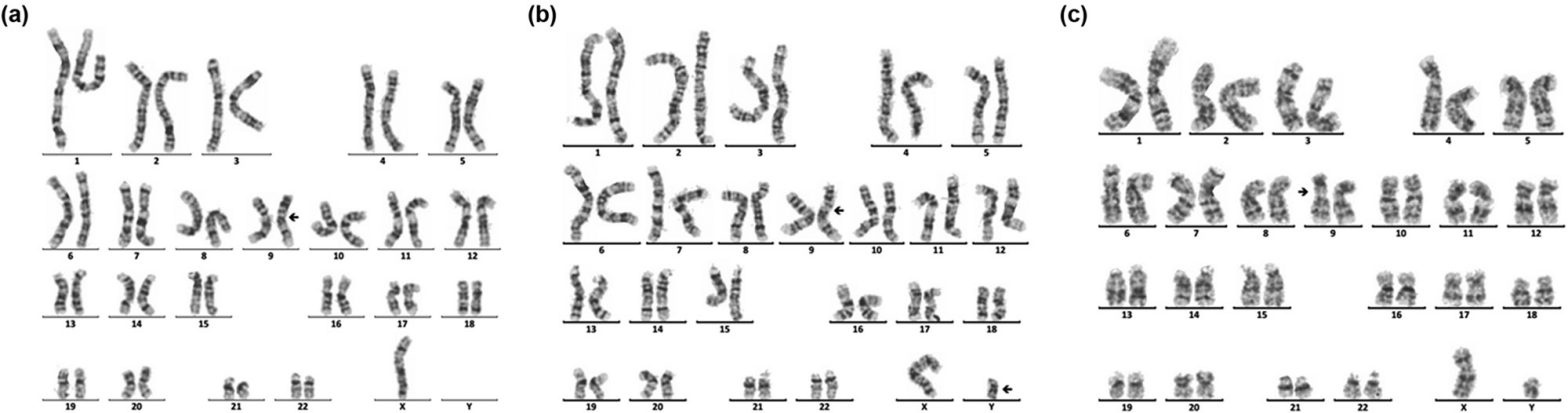
Karyotype of 45,X,inv(9)(p11q13)[72]/46,X,dic(Y)(q11.23),inv(9)(p11q13)[10]/46,XY,inv(9)(p11 q13)[4]. The arrows show abnormal chromosomes. (a) 45,X,inv(9)(p11q13). (b) 46,X,dic(Y)(q11.23), inv(9)(p11q13). (c) 46,XY,inv(9) (p11q13).

**Figure 3:**
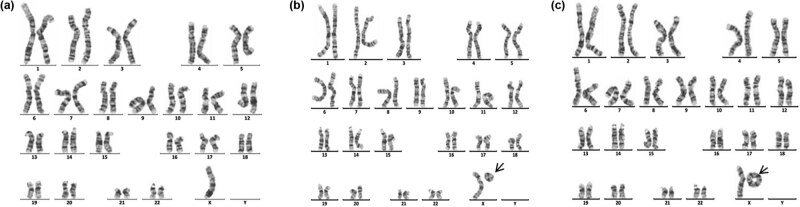
Karyotype of 45,X[120]/46,X,r(X)(p22.2q22.2)[12]/46,X,rdup(X)(p22.2q22.2)[4]. The arrows show abnormal chromosomes. (a) 45,X. (b) 46,X,r(X)(p22.2q22.2). (c) 46,X,rdup(X)(p22.2q22.2).

**Figure 4:**
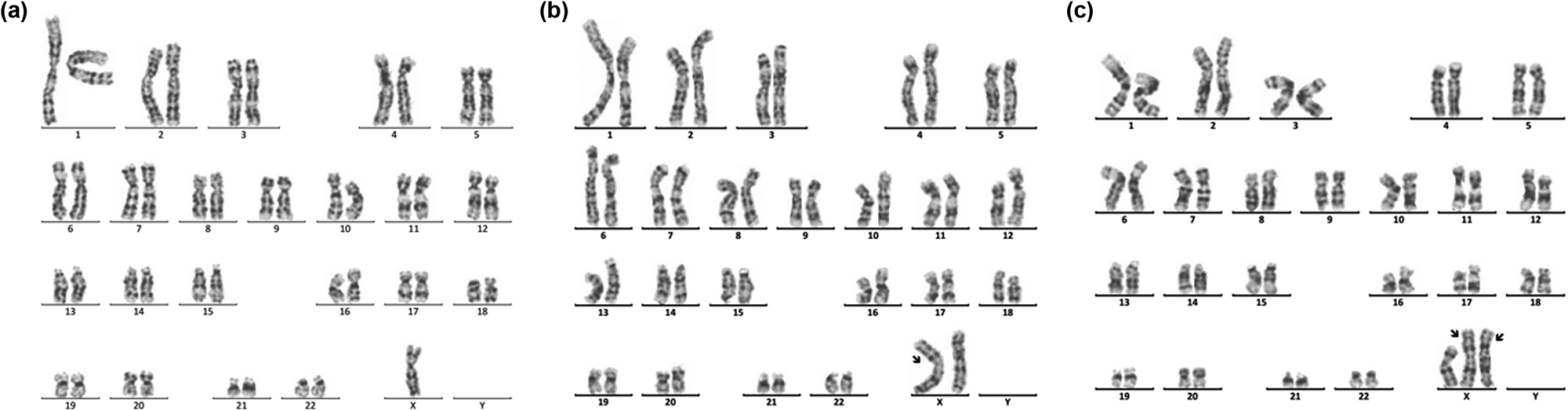
Karyotype of 46,X,idic(X)(p11.2)[92]/45,X[9]/47,X,idic(X)(p11.2),idic(X)(p11.2)[5]. The arrows show abnormal chromosomes. (a) 45,X. (b) 46,X,idic(X)(p11.2). (c) 47,X,idic(X)(p11.2),idic(X)(p11.2).

**Figure 5:**
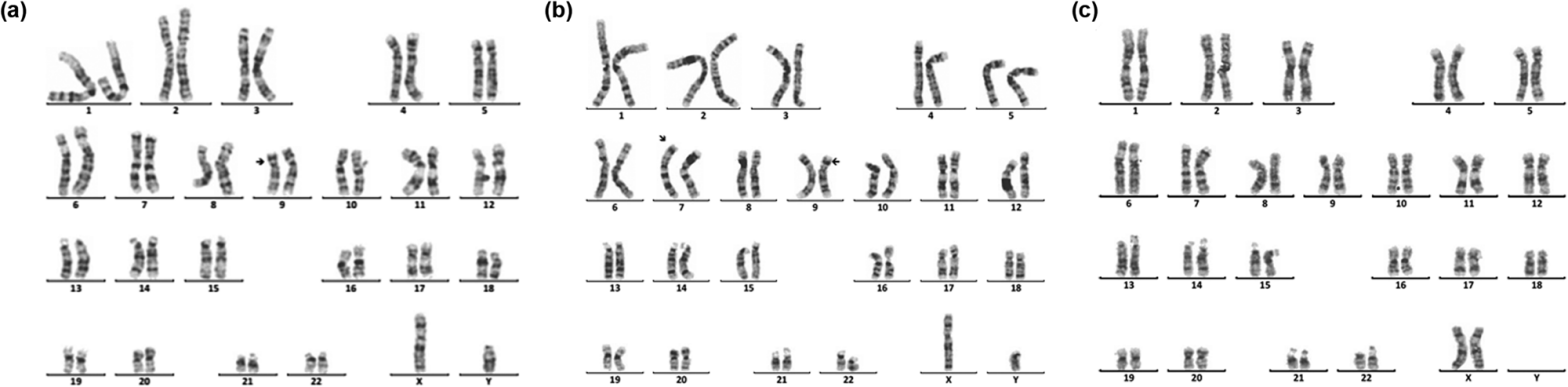
Karyotype of 46,XY,der(9)t(7;9)(p15;p22)pat. The arrows show abnormal chromosomes. (a) The karyotype of the patient. (b) The karyotype of the patient’s father. (c) The karyotype of the patient’s mother.

**Figure 6:**
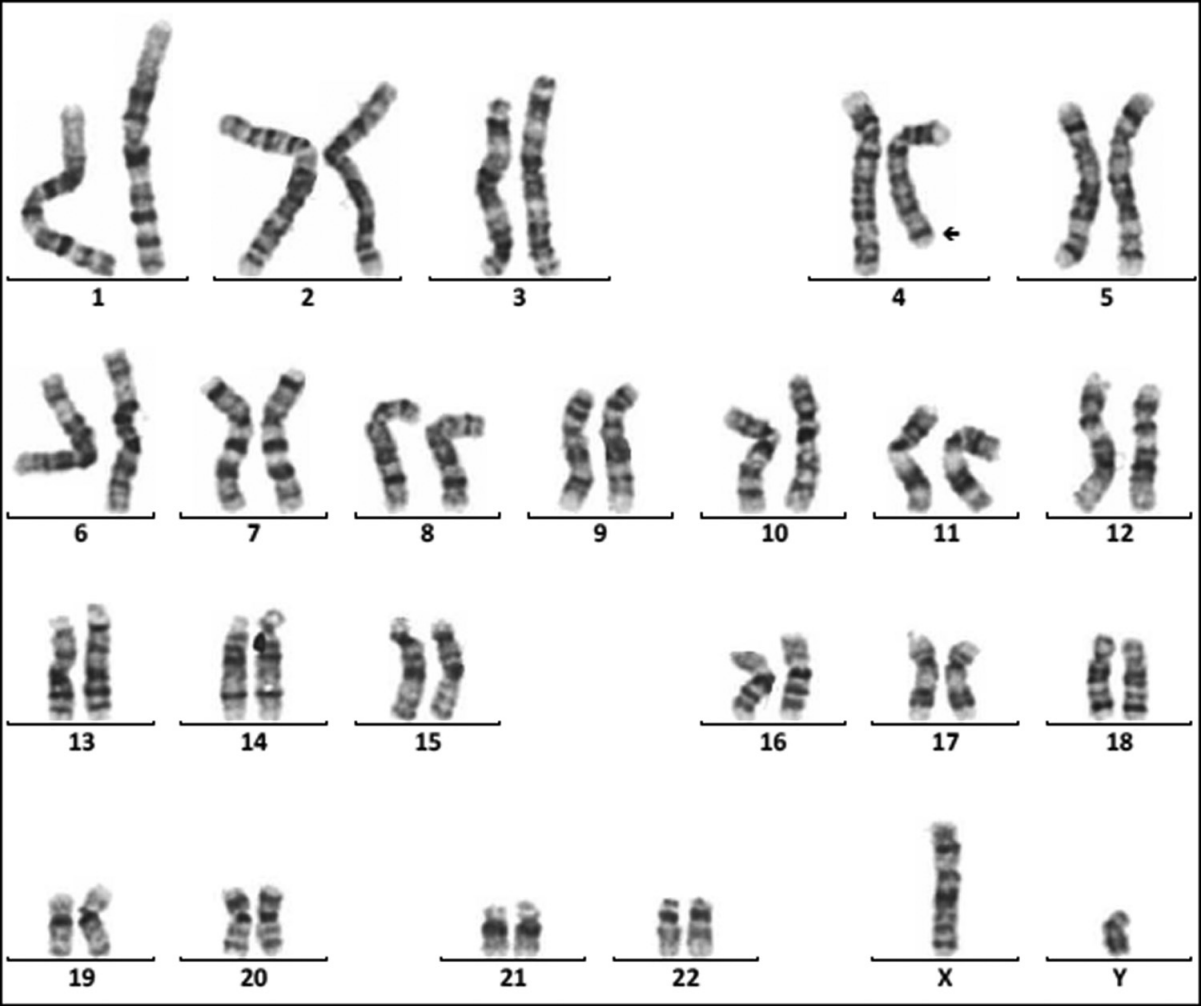
Karyotype of 46,XY,del(4)(q33). The arrows show abnormal chromosomes.

**Figure 7:**
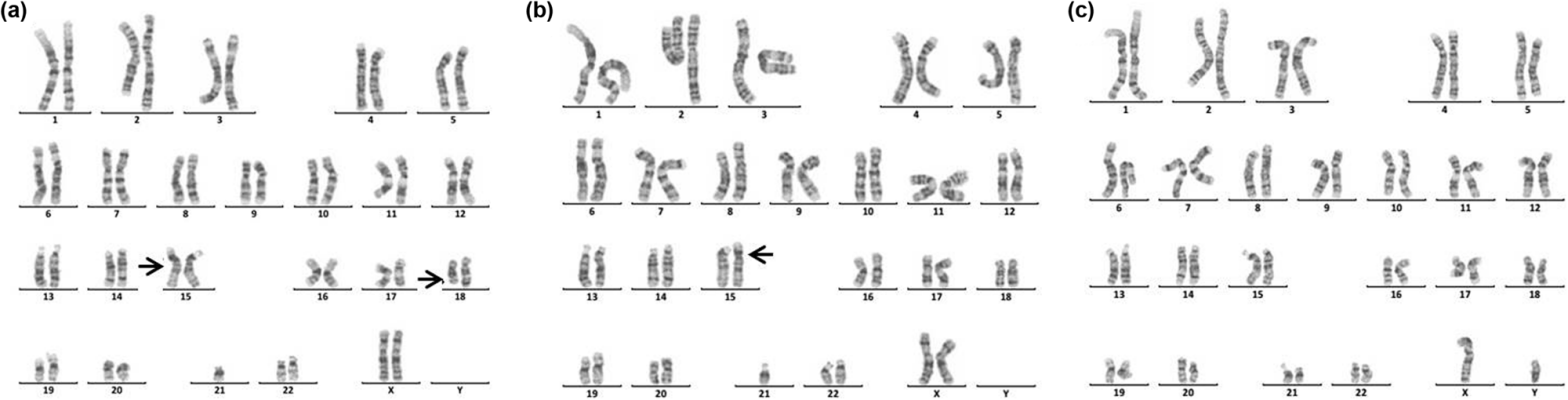
Karyotype of 45,XX,der(15;21)(q10;q10)mat,del(18)(q21). The arrows show abnormal chromosomes. (a) The karyotype of the patient. (b) The karyotype of the patient’s mother. (c) The karyotype of the patient’s father.

Sixteen cases of rare abnormal karyotype were detected, including complex Tri21 (Figure 1), complex TS (Figures 2–4), partial monomer and partial trisomy (Figures 5), 4p-syndrome (Figure 6), 18q-syndrome (Figure 7), and 5p-syndrome.

## 4 Discussion

Chromosomal diseases are often associated with growth retardation, mental retardation, and multiple malformations of organs, which are congenital diseases caused by chromosome number or structural abnormalities. In our study, 181 cases with abnormal karyotype were detected, and the abnormal rate was 41.51% (181/436), which indicated that chromosomal abnormalities were one of the important causes in children with dysplasia.

Intellectual disability (80.66%, 146/181) and dysmorphology (71.82%, 130/181) are the main clinical symptoms of 181 children with chromosomal abnormalities. As known, Tri21 is the most common chromosomal disorder, which implicated with intellectual disability and dysmorphology. The prevalence of Tri21 ranges from 1/700 to 1/2,000 in different ethnic populations investigated [17]. The cause of Tri21 is related to a variety of factors, such as increased reproductive age in women (>35 years old), degeneration of ovarian function, and genetic susceptibility [18,19]. In our study, 136 cases of Tri21 were detected (31.19%, 136/436), including 89 male and 47 female (1.89:1, 89/47). Among them, 130 cases were standard Tri21 (95.59%, 130/136), 5 cases were Robertson translocation Tri21 (3.79%, 5/132), and 1 case was a chimerical type (0.74%, 1/136), which is consistent with previous studies [20,21,22]. In 136 cases of Tri21, about two-thirds of pregnant women did not receive maternal serum screening for DS, and 16 pregnant women’s results of maternal serum screening for DS were high risk, but they did not receive a prenatal diagnosis. Therefore, enhancing people’s awareness of the importance of prenatal screening and genetic counseling in eugenics may be an important means of reducing birth in children with Tri21. In addition, one case with 46,XX,der(21;21)(q10;q10),t(1;12)(q43;p12.1), inv(15)(q13q24) (Figure 1), which is a rare complex Tri21 karyotype, was detected. So, this case is especially helpful to supplement the karyotype diversity of patients with Tri21.

SDS and short stature are the two other major clinical symptoms of 181 children with chromosomal abnormalities. As known, chromosomal abnormalities are one of the important causes of gonadal dysgenesis and physical retardation. In this study, 28 cases of sex chromosome abnormalities were detected, among which 20 cases were TS, and 5 cases were 46,X,i(X)(q10). The main manifestations of TS patients were immature uterus or no uterus, streak ovary or no ovary, primary amenorrhea, breast dysplasia, and short stature, and the main clinical symptoms of the patients with 46,X,i(X)(q10) were short stature and primary amenorrhea. These results suggested the necessity of two intact X chromosomes in the normal growth and development of a female. A single X chromosome in females randomly becomes inactive, but not all genes on which are transcriptionally silenced. Still, 15–20% of genes on inactive X chromosome remain operative and escape from X inactivation [23]. Moreover, normal female development is supported by a double dose of several specific genes on the X chromosome, such as the short stature homeobox (SHOX) gene, which is located at the Xp22, determines the height [24]. So, the haploinsufficiency of SHOX may be the main cause of short stature in patients. Dysmorphology and short stature caused by an X chromosome abnormality are more sensitive to estrogen before 12 years old. So, these kinds of patients need to receive treatment in the early stage. However, in this study, the average age of the patient with TS was11.94, greater than 6.6, which was reported by Massa et al. [25]. Consequently, the growth and development of children should be highly concerned during the preschool period. In addition, another three rare complex karyotypes were detected. One karyotype was 45,X,inv(9)(p11q13)[72]/46,X,dic(Y)(q11.23),inv(9)(p11q13)[10]/46,XY,inv(9)(p11q13)[4] (Figure 2), the other was 45,X[120]/46,X,r(X)(p22.2q22.2)[12]/46,X, rdup(X)(p22.2q22.2)[4] (Figure 3), and the third was 46,X,idic(X)(p11.2)[92]/45,X [9]/47,X,idic(X)(p11.2), idic(X)(p11.2)[5] (Figure 4).

Chromosome monomer or trisomy is one of the important causes of congenital malformation in children. Monomer 9p is a rare condition accompanied by trigonocephaly, facial dysmorphism, and developmental delay [26,27]. In this study, two cases with monosomy 9p were described. The karyotype of one patient was 46,XY,der(9)t(2;9)(p25;p22)mat, which presented with small ears, low ear position, small jaw, wide breast distance, short chest, long fingers and toes, inability to extend the second joint of the middle finger of both hands, special clenched fist posture (index finger pressed on the middle finger, little finger pressed on the ring finger) [28]. The karyotype of another patient was 46,XX,der(9)t(7;9)(p15;p22)pat (Figure 5). Studies have shown that patients with der(9)t(7;9) are mostly characterized by developmentally delayed psychomotor retardation and generalized developmental deficits (Table 3). In this study, the patient presents as developmentally delayed and psychomotor retardation, according to the previous report.

**Table 3:** Clinical symptoms of the patient with der(9)t(7;9)

Karyotype	Abnormal phenotype	Authors
46, XY, der(9) t(7;9)(p15;p22) pat	Seizure, developmentally delayed, delayed myelination, and widened brain extracellular space	Zhong et al. [29]
46, XY,t(7;9)(p22;q22)mat	Hypotrophic, full and wavy hair; a prominent forehead (middle facial part); microcephaly; low-set abnormal ears; hypertelorism; narrow, short eye slits; antimongoloid eye slant; broad, flat nasal bridge; bulbous nasal tip; microretrognathia; high palate; macrostomia; short neck; hollow stomach; short upper and lower extremities; bilateral clinodactyly of second and fifth fingers; thumbs and first toes are positioned far from other fingers (sandal gap); hypoplasia of toes nails; single transverse palmar crease; hypoplastic aortic arch; and hypoplastic lungs	Manvelyan et al. [30]
46, XY,der(9)t(7;9)(p21.2;p23.5)	Bilateral choanal atresia, growth delay, marked psychomotor retardation, hydronephrosis, muscular hypotonia	Back et al. [31]
46,XY, der(9),t(7;9)(q31.1;p23)pat	Generalized mild dysmorphic, heart failure, and hydrocephalus, sex reversal	Crocker et al. [32]
46,XX,der(9)t(7;9)(p15.3;p24)	Psychomotor retardation, upward slant of palpebral fissures, and dolichomesophalangy	Teebi et al. [33]
46,XX,der(9)t(7;9) (q35;q22.2)	Hypoplasia of the cerebellar vermis, dilated foramen Magendii, and dilatation of the cisterna magna	von Kaisenberg et al. [34]
46, XX, der(9)t(7;9)(p15; p24)	Generalized developmental deficits, a high and large forehead, hypertelorism, and broad nasal bridge, hypothyroidism, obesity, cerebral palsy, severe mental retardation	Kozma et al. [35]
46,XX,der(9)t(7;9)(p15;p22)pat	Developmental retardation and mental retardation	Present case

Chromosome 4q deletion syndrome (4q-syndrome) is a rare condition, with an estimated incidence of 1 in 100,000 and the death rate was about 28% [36,37]. Although the clinical symptoms of patients with 4q-syndrome are complex and diverse (Table 4), through statistical analysis of the clinical symptoms of 101 patients, Strehle et al. found that craniofacial (99%), digital (88%), skeletal (54%), and cardiac (50%) were the most common anomalies [37]. Keeling et al. have reported that the critical region involved in the 4q terminal deletion syndrome may be 4q33 [43]. In this study, we reported a 16-month-old boy with 4q33-qter deletion (Figure 6). The patient presents with cleft palate, micrognathia, sydney line of the left hand, and developmental delay. Our results further support the idea that cleft palate-related genes might be located at 4q33 [43]. In addition, although most patients with 4q-syndrome have de novo deletions, familial cases have been reported, suggesting a high risk of recurrence of 4q-syndrome [47,48]. So, for these patients, prenatal diagnosis is necessary.

**Table 4:** Clinical symptoms of the patient with 4q-syndrome

Karyotype	Abnormal phenotype	Authors
Deletion of the segment 4q22.1-q23	Slight developmental delay, mild dysmorphic features	Strehle et al. [38]
Deletion of segment 4q28.3-q31.23	Growth failure, developmental delay, ventricular septum defect in the subaortic region, patent foramen ovale and patent ductus arteriosus, vascular malformation of the lung, dysgenesis of the corpus callosum and craniofacial dysmorphism	Duga et al. [39]
Deletion of segment 4q31-qter	Craniofacial dysmorphism, skeletal anomalies, ocular findings, and cardiac defect	Sandal et al. [40]
Deletion of segment q31.2-q35.2	Craniofacial hypoplasia of left side of face, ipsilateral ptosis, erythroderma, and bilateral thumb anomalies	Kuldeep et al. [41]
Deletion of segment 4q31.21-q31.23	Pseudohypoaldosteronism	Pritchard et al. [42]
Deletion of segment 4q31.3-qter	Complex CHD	Strehle et al. [38]
Deletion of segment 4q32-q34	Mild developmental delay; a left ulnar ray defect with absent ulna and associated metacarpals, carpals, and phalanges; and a right ulnar nerve hypoplasia	Keeling et al. [43]
Deletion of segment 4q32.3-q34.3	Congenital heart defects	Xu et al. [44]
Deletion of segment 4q33-qter	Mildly dysmorphic, heart failure, and hypercalcaemia	Strehle et al. [45]
Deletion of segment 4q33-qter	Cleft palate, micrognathia, sydney palm of left hand, and developmental delay	Present case
Deletion of segment 4q34.2-qter	CHD, submucosal cleft palate, hypernasal speech, learning difficulties, and right fifth finger anomaly manifestations	Tsai et al. [46]
Deletion of segment 4q34.3-qter	Asymptomatic cor triatriatum sinister	Marcì et al. [47]

Deletion of the long arm of chromosome 18 (18q-) is relatively common among cytogenetic abnormality, which occurs incidentally in approximately 1 in 40,000 live births [49]. 18q-syndrome is characterized by a wide range of phenotypic abnormalities related to the size of the deletion and the position of breakpoints. The common clinical features of the 18q-syndrome are growth retardation, mental retardation, microcephaly, facial dysmorphisms, ear atresia, abnormal bone development, CHD, cerebral white matter abnormalities, and immature myelin formation [50,51,52]. In this study, the patient with a rare karyotype of 45,XX,der(15;21)(q10;q10)mat, del(18)(q21) (Figure 7) presented with mental retardation, unclear diction, facial dysmorphisms, abnormal bone development, the little finger end bending, poor balance ability, and unsteady walking. In addition, the patient also suffered from type I diabetes and Hashimoto’s thyroiditis, which were rarely reported. Therefore, this case is especially helpful in supplementing the phenotypic diversity of patients with 18q.

## 5 Conclusion

Chromosome abnormality is one of the important causes of dysplasia in children, and prenatal screening and diagnosis could play a great significance in preventing dysplasia in children. In addition, the retrospective analysis of the rare case is valuable for clinical diagnosis and risk assessment of recurrence.

## References

[1] Wu XL, Li R, Fu F, Pan M, Han J, Yang X, et al. Chromosome microarray analysis in the investigation in children with congenital heart disease. BMC Pediatr. 2017;17:117. 10.1186/s12887-017-0863-3 PMC5418813 28472932

[2] Hartman RJ, Rasmussen SA, Botto LD, Riehle-Colarusso T, Martin CL, Cragan JD, et al. The contribution of chromosomal abnormalities to congenital heart defects: a population-based study. Pediatr Cardiol. 2011;32:1147–57. 10.1007/s00246-011-0034-5 21728077

[3] Huang Z, Sun Y, Fan Y, Wang L, Liu H, Gong Z, et al. Genetic evaluation of 114 Chinese short stature children in the next generation era: a single center study. Cell Physiol Biochem. 2018;49:295–305. 10.1159/000492879 30138938

[4] Britto IS, Regina Silva Herbest S, Tedesco GD, Drummond CL, Bussamra LC, Araujo Júnior E, et al. Prenatal diagnosis of a fetus with ring chromosomal 15 by two-and three-dimensional ultrasonography. Case Rep Obstet Gynecol. 2014;2014:495702. 10.1155/2014/495702 PMC4217343 25389503

[5] Szabó A, Czakó M, Hadzsiev K, Duga B, Bánfai Z, Komlósi K, et al. Small supernumerary marker chromosome 15 and a ring chromosome 15 associated with a 15q26.3 deletion excluding the IGF1R gene. Am J Med Genet A. 2018;176:443–9. 10.1002/ajmg.a.38566 29226546

[6] Paz-Y-Miño C, Guevara-Aguirre J, Paz-Y-Miño A, Velarde F, Armendáriz-Castillo I, Yumiceba V, et al. Ring chromosome 15-cytogenetics and mapping arrays: a case report and review of the literature. J Med Case Rep. 2018;16(12):340. 10.1186/s13256-018-1879-5 PMC6238305 30442194

[7] Ribeiro Dias Barroso C, Silveira Gomes L, Abrantes Silvestre V, Yamada Utagawa C. Cutis tricolor parvimaculata in ring chromosome 15 syndrome: a case report. Pediatr Dermatol. 2018;35:e204–5. 10.1111/pde.13497 29658137

[8] Chen CP, Lin SP, Chern SR, Wu PS, Chen YN, Chen SW, et al. Molecular cytogenetic characterization of mosaicism for a small supernumerary marker chromosome derived from chromosome 8 or r(8)(: p11.22 → q11.21:) in an 18-year-old female with short stature, obesity, attention deficit hyperactivity disorder, and intellectual disability. Taiwan J Obstet Gynecol. 2016;55:856–60. 10.1016/j.tjog.2016.08.003 28040133

[9] Chen CP, Lin SP, Lin YH, Chern SR, Wu PS, Chen YN, et al. Molecular cytogenetic characterization of mosaicism for a small Supernumerary marker chromosome derived from chromosome 8 or r(8)(: p12 → q13.1:) associated with phenotypicabnormalities. Taiwan J Obstet Gynecol. 2016;55:852–5. 10.1016/j.tjog.2016.08.002 28040132

[10] Farcas S, Erdelean D, Szekely FA, Navolan D, Andreescu N, Cioca A. A rare case of partial trisomy 8q24.12-q24.3 and partial monosomy of 8q24.3: prenatal diagnosis and clinical findings. Taiwan J Obstet Gynecol. 2019;58:36–9. 10.1016/j.tjog.2018.11.005 30638476

[11] Chen CP, Chen M, Ko TM, Ma GC, Tsai FJ, Tsai MS, et al. Prenatal diagnosis and molecular cytogenetic characterization of a small supernumerary marker chromosome derived from chromosome 8. Taiwan J Obstet Gynecol. 2010;49:500–5. 10.1016/S1028-4559(10)60104-0 21199754

[12] Chen CP, Chang SD, Su YN, Chen M, Chern SR, Su JW, et al. Rapid positive confirmation of mosaicism for a small supernumerary marker chromosome as r(8) by interphase fluorescence in situ hybridization, quantitative fluorescent polymerase chain reaction, and array comparative genomic hybridization on uncultured amniocytes in a pregnancy with fetal pyelectasis. Taiwan J Obstet Gynecol. 2012;51:405–10. 10.1016/j.tjog.2012.07.016 23040926

[13] Shao HY, Miao ZY, Liu XY, Hou XF, Wu H. Molecular cytogenetic characterization of mosaicism for a small supernumerary marker chromosome derived from chromosome 8 associated with congenital hypoplasia of the tongue and review of the literature. Taiwan J Obstet Gynecol. 2020;59:323–6. 10.1016/j.tjog.2020.01.025 32127158

[14] Spazzapan P, Arnaud E, Baujat G, Nizon M, Malan V, Brunelle F, et al. Clinical and neuroradiological features of the 9p deletion syndrome. Childs Nerv Syst. 2016;32:327–35. 10.1007/s00381-015-2957-2 26597681

[15] Hou QF, Wu D, Chu Y, Liao SX. Clinical findings and molecular cytogenetic study of de novo pure chromosome 9p deletion: Pre- and postnatal diagnosis. Taiwan J Obstet Gynecol. 2016;55:867–70. 10.1016/j.tjog.2016.11.001 28040136

[16] Tawn EJ, Curwen GB, Jonas P, Riddell AE, Hodgson L. Chromosome aberrations determined by sFISH and G-banding in lymphocytes from workers with internal deposits of plutonium. Int J Radiat Biol. 2016;92:312–20. 10.3109/09553002.2016.1152414 PMC4898148 27043761

[17] Wu H, Miao ZY, Hou XF, Liu XY, Shao HY. Prenatal diagnosis of low-level mosaicism for trisomy 21 with rare karyotype detected by noninvasive prenatal testing. Taiwan J Obstet Gynecol. 2017;56:703–5. 10.1016/j.tjog.2017.08.027 29037564

[18] Yamamot T, Shimojima K, Nishizawa T, Matsuo M, Ito M, Imai K. Clinical manifestations of the deletion of Down syndrome critical region including DYRKlA and KCNJ6. Am J Med Genet A. 2011;155A:113–9. 10.1002/ajmg.a.33735 21204217

[19] Grebe C, Klingebiel TM, Graus P, Toischer K, Didié M, Jacobshagen C, et al. Enhanced expression of DYRKlA in cardiomyocytes inhibits acute NFAT activation but does not prevent hypertrophy in vivo. Cardiovase Res. 2011;90:521–8. 10.1093/cvr/cvr023 21273244

[20] Zhao W, Chen F, Wu M, Jiang S, Wu B, Luo H, et al. Postnatal identification of trisomy 21: an overview of 7,133 postnatal trisomy 21 cases identified in a diagnostic reference laboratory in China. PLoS One. 2015;10:e0133151. 10.1371/journal.pone.0133151 PMC4503670 26176847

[21] Balkan M, Akbas H, Isi H, Oral D, Turkyilmaz A, Kalkanli S, et al. Cytogenetic analysis of 4216 patients referred for suspected chromosomal abnormalities in Southeast Turkey. Genet Mol Res. 2010;9:1094–103. 10.4238/vol9-2gmr827 20568054

[22] Mandava S, Koppaka N, Bhatia V, Das BR. Cytogenetic analysis of 1572 cases of down syndrome: a report of double aneuploidy and novel findings 47,XY,t(14;21)(q13;q22.3)mat, + 21 and 45,XX,t(14;21)in an Indian population. Genet Test Mol Biomarkers. 2010;14:499–504. 10.1089/gtmb.2009.0167 20642367

[23] Carrel L, Cottle AA, Goglin KC, Willard HF. A first-generation X inactivation profile of the human X chromosome. Proc Natl Acad Sci USA. 1999;96:14440–4. 10.1073/pnas.96.25.14440 PMC24455 10588724

[24] Seo GH, Kang E, Cho JH, Lee BH, Choi JH, Kim GH, et al. Turner syndrome presented with tall stature due to overdosage of the SHOX gene. Ann Pediatr Endocrinol Metab. 2015;20:110–3. 10.6065/apem.2015.20.2.110 PMC4504991 26191517

[25] Massa G, Verlinde F, De Schepper J, Thomas M, Bourguignon JP, Craen M, et al. Trends in age at diagnosis of turner syndrome. Arch Dis Child. 2005;90:267–8. 10.1136/adc.2004.049817 PMC1720288 15723912

[26] León-Carlos NY, García-Delgado C, Morales-Jiménez AB, Serrano-Bello C, Cervantes A, Morán Barroso VF. Monosomy 9p24 in two non-related patients as result of a translocation (2;9). Arch Argent Pediatr. 2018;116:e603–8. 10.5546/aap.2018.e603 30016040

[27] Spazzapan P, Arnaud E, Baujat G, Nizon M, Malan V, Brunelle F, et al. Clinical and neuroradiological features of the 9p deletion syndrome. Childs Nerv Syst. 2016;32:327–35. 10.1007/s00381-015-2957-2 26597681

[28] Miao ZY, Shao HY, Wu H. One case of 46,XY,der(9)t(2;9)(p25;p22)mat. Chin J Med Genet. 2016;1:47.

[29] Zhong M, Dong Y, Li M, Yao. H. Infantile spasms in a boy with an abnormal karyotype (46, XY, der(9)t(7;9)(p15;p22)pat). Neurol India. 2014;62:189–91. 10.4103/0028-3886.132393 24823731

[30] Manvelyan M, Simonyan I, Hovhannisyan G, Aroutiounian R, Hamid AB, Liehr T. A new case of a complex small supernumerary marker chromosome: a der(9)t(7;9)(p22;q22) due to a maternal balanced rearrangement. J Pediatr Genet. 2015;4:199–200. 10.1055/s-0035-1565270 PMC4906527 27617132

[31] Back E, Jung C, Zeitler S, Schempp W. De novo duplication of 7pter-- > p21.2 and deletion of 9pter-- > p23.5: clinical and cytogenetic diagnosis. Clin Genet. 1997;51:56–60. 9084937

[32] Crocker M, Coghill SB, Cortinho. R. An unbalanced autosomal translocation (7;9) associated with feminization. Clin Genet. 1988;34:70–3. 10.1111/j.1399-0004.1988.tb02618.x 3409542

[33] Teebi AS, Gibson L, McGrath J, Meyn MS, Breg WR, Yang-Feng TL. Molecular and cytogenetic characterization of 9p- abnormalities. Am J Med Genet. 1993;46:288–92. 10.1002/ajmg.1320460310 8488873

[34] von Kaisenberg CS, Caliebe A, Krams M, Hackelöer BJ, Jonat W. Absence of 9q22-9qter in trisomy 9 does not prevent a Dandy-Walker phenotype. Am J Med Genet. 2000;95:425–8. 11146460

[35] Kozma C, Haddad BR, Meck JM. Trisomy 7p resulting from 7p15;9p24 translocation: report of a new case and review of associated medical complications. Am J Med Genet. 2000;91:286–90. 10.1002/(sici)1096-8628(20000410)91:4<286::aid-ajmg9>3.0.co;2-2 10766985

[36] Strehle EM, Yu L, Rosenfeld JA, Donkervoort S, Zhou Y, Chen TJ, et al. Genotype-phenotype analysis of 4q deletion syndrome: proposal of a critical region. Am J Med Genet A. 2012;158A(9):2139–51. 10.1002/ajmg.a.35502 22847869

[37] Strehle EM, Bantock HM. The phenotype of patients with 4q-syndrome. Genet Couns. 2003;14(2):195–205. 12872814

[38] Strehle EM, Gruszfeld D, Schenk D, Mehta SG, Simonic I, Huang T. The spectrum of 4q- syndrome illustrated by a case series. Gene. 2012;506(2):387–91. 10.1016/j.gene.2012.06.087 22771923

[39] Duga B, Czako M, Komlosi K, Hadzsiev K, Torok K, Sumegi K, et al. Deletion of 4q28.3-31.23 in the background of multiple malformations with pulmonary hypertension. Mol Cytogenet. 2014;7:36. 10.1186/1755-8166-7-36 PMC4066825 24959202

[40] Sandal G, Ormeci AR, Oztas S. De novo terminal 4q deletion syndrome with new ocular findings in Turkish twins: case report. Genet Couns. 2013;24(2):217–22. 24032293

[41] Kuldeep CM, Khare AK, Garg A, Mittal A, Gupta L. Terminal 4q deletion syndrome. Indian J Dermatol. 2012;57(3):222–4. 10.4103/0019-5154.96203 PMC3371530 22707778

[42] Pritchard AB, Ritter A, Kearney HM, Izumi. K. Interstitial 4q deletion syndrome including NR3C2 causing pseudohypoaldosteronism. Mol Syndromol. 2020;10(6):327–31. 10.1159/000505279 PMC6995944 32021607

[43] Keeling SL, Lee-Jones L, Thompson P. Interstitial deletion 4q32-34 with ulnar deficiency: 4q33 may be the critical region in 4q terminal deletion syndrome. Am J Med Genet. 2001;99(2):94–8. 10.1002/1096-8628(2000)9999:999<00::aid-ajmg1134>3.0.co;2-d 11241465

[44] Xu W, Ahmad A, Dagenais S, Iyer RK, Innis JW. Chromosome 4q deletion syndrome: narrowing the cardiovascular critical region to 4q32.2-q34.3. Am J Med Genet A. 2012;158A(3):635–40. 10.1002/ajmg.a.34425 22302627

[45] Strehle EM, Ahmed OA, Hameed M, Russell A. The 4q-syndrome. Couns. 2001;12(4):327–39. 11837601

[46] Tsai CH, Van Dyke DL, Feldman GL. Child with velocardiofacial syndrome and del (4)(q34.2): another critical region associated with a velocardiofacial syndrome-like phenotype. Am J Med Genet. 1999;82(4):336–39. 10051168

[47] Marcì M, Guarina A, Castiglione MC, Sanfilippo N. Different cardiac anomalies in mother and son with 4q-syndrome. Genet. 2015;2015:932651. 10.1155/2015/932651 PMC4568327 26417463

[48] Lall M, Puri R, Saviour P, Verma I. A familial deletion 4q syndrome: an outcome of a paracentric inversion. Indian J Hum Genet. 2012;18(2):238–40. 10.4103/0971-6866.100780 PMC3491302 23162304

[49] Cody JD, Ghidoni PD, DuPont BR, Hale DE, Hilsenbeck SG, Stratton RF, et al. Congenital anomalies and anthropometry of 42 individuals with deletions of chromosome 18q. Am J Med Genet. 1999;85(5):455–62. 10.1002/(sici)1096-8628(19990827)85:5<455::aid-ajmg5>3.0.co;2-z 10405442

[50] Mark PR, Radlinski BC, Core N, Fryer A, Kirk EP, Haldeman-Englert CR. Narrowing the critical region for congenital vertical talus in patients with interstitial 18q deletions. Am J Med Genet A. 2013;161A(5):1117–21. 10.1002/ajmg.a.35791 23495172

[51] Perry BP, Sebold C, Hasi M, Heard P, Carter E, Hill A, et al. Sensorineural hearing loss in people with deletions of 18q: hearing in 18q-. Otol Neurotol. 2014;35(5):782–86. 10.1097/MAO.0000000000000363 PMC4170734 24662633

[52] Tassano E, Severino M, Rosina S, Papa R, Tortora D, Gimelli G, et al. Interstitial de novo 18q22.3q23 deletion: clinical, neuroradiological and molecular characterization f a new case and review of the literature. Mol Cytogenet. 2016;9:78. 10.1186/s13039-016-0285-1 PMC5057431 27766118

